# Making a difference: describing and evaluating the impact of the Dutch CardioVascular Alliance

**DOI:** 10.1007/s12471-025-01975-y

**Published:** 2025-08-14

**Authors:** Sopany Saing, Jolien W. Roos-Hesselink, Astrid Schut, Thea van Asselt, Rebecca Abma-Schouten, Margien G. S. Boels, Naomi Tramper, Clara van Ofwegen-Hanekamp, Robert Willemsen, Michelle M. A. Kip, Hendrik Koffijberg

**Affiliations:** 1 Health Technology & Services Research, Faculty of Behavioural Management and Social Sciences, University of Twente, Enschede, The Netherlands; 2 Dutch CardioVascular Alliance (DCVA), Utrecht, The Netherlands; 3 Erasmus Medical Centre Rotterdam, Rotterdam, The Netherlands; 4 Werkgroep Cardiologische centra Nederland (WCN), Utrecht, The Netherlands; 5 Department of Epidemiology & Department of Health Sciences, University of Groningen, University Medical Center Groningen, Groningen, The Netherlands; 6 Hartstichting (Dutch Heart Foundation), The Hague, The Netherlands; 7 Diakonessenhuis Utrecht, Utrecht, The Netherlands; 8 Maastricht University, Maastricht, The Netherlands

**Keywords:** Health technology assessment, Burden of disease, Cardiovascular disease, Alliance, Collaboration

## Abstract

**Introduction:**

In 2018, the Dutch CardioVascular Alliance (DCVA), a collaboration between 24 partners in the cardiovascular field, expressed the ambition to reduce the cardiovascular disease (CVD) burden in the Netherlands by 25% in 2030. This project aimed to evaluate the extent to which the activities within the DCVA contribute to a reduction in the burden defined as morbidity and mortality combined.

**Methods:**

The role and potential impact of the DCVA was assessed. Three assessments were conducted: 1) to determine the potential impact of consortia (*n* = 32) using a checklist; 2) to estimate the potential health benefit (quality-adjusted life years, (QALYs)) and cost savings from a snapshot of consortia (*n* = 4).

**Results:**

Most of the consortia focused on treatment (31%), followed by secondary prevention/monitoring (23%) and diagnosis (23%). Almost all consortia (*n* = 31) aim to reduce morbidity and two-thirds (*n* = 21) aim to reduce mortality. The four consortia evaluated were Check@Home, LoDoCo2, CONTRAST 2.0 and IMPRESS, with pathways in screening, treatment, treatment and diagnosis, respectively. The total estimated cumulative QALYs gained (from 2023 to 2030) were 1,694, 362, 2,783, and 3,655 respectively.

**Discussion:**

Although it is impossible to estimate the full impact of the DCVA itself, the presented checklist and analyses may increase awareness of the different DCVA activities, roles, and consortia. Existing HTA methods can support the exploration of the potential impact generated by each consortium within the DCVA. The current portfolio of DCVA consortia contributes extensively to the DCVA goal of reducing the CVD burden, provided there is effective support for the adoption and implementation of innovations.

**Supplementary Information:**

The online version of this article (10.1007/s12471-025-01975-y) contains supplementary material, which is available to authorized users.

## Introduction

Cardiovascular diseases (CVD) are among the leading causes of morbidity and mortality worldwide. CVD accounts for approximately one-third of deaths globally, with 18 million deaths from CVD in 2017, representing an increase of 21% compared to 2007 [1]. It is estimated that this trend will continue and that annual mortality will grow to almost 24 million by 2030 [2]. With a population of 17.9 million in 2024 [3], there are currently 1.7 million people in the Netherlands known to have CVD [4] (at a cost for 6.8 billion Euros in 2019 [5]), and without major intervention this number is expected to increase sharply in the coming years to 2.6 million in 2030 [6, 7].

In 2018, leading organisations in the field, including representatives of patients, academia, healthcare professionals, health funds, research funders, and industry united in the Dutch CardioVascular Alliance (DCVA). The DCVA facilitates and stimulates collaboration between its partners, who have jointly set a goal to reduce the burden of CVD by 25% by 2030. In 2023, half of the time set to achieve the goal had passed, so it was an appropriate time to reflect on the process and activities of these partners within the DCVA. Although the impact of partners within the DCVA cannot be estimated compared to their impact without the DCVA, and formal methods to quantitatively evaluate the value of scientific and policy collaboration are lacking, society has an active interest in learning about activities and potential impact from partners within the DCVA. This reflection aimed to describe the activities of DCVA partners and the role of the DCVA and to investigate how these are likely to contribute to this aim, to explore if the DCVA and partners are heading in the right direction. In addition, this enhances awareness of all stakeholders in society to understand *how* DCVA projects may reduce the cardiovascular disease burden and align with the national aim to add healthy life years to citizens’ lives [8].

**Fig. 1:**
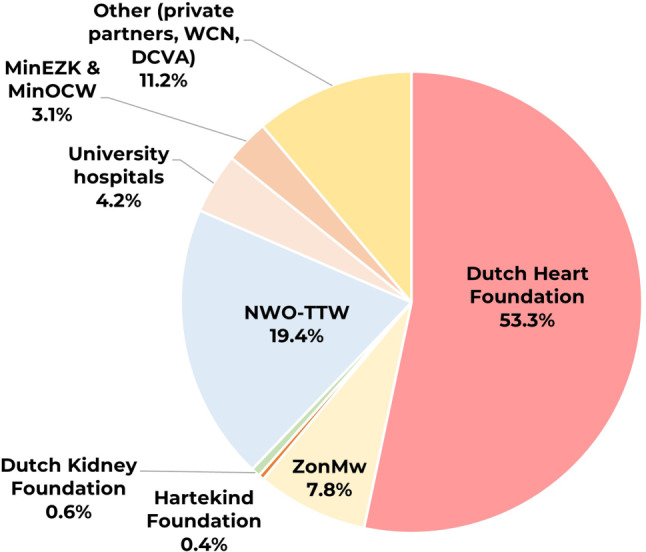
In cash contributions to projects supported by the DCVA (% of total). *DCVA* Dutch Cardiovascular Alliance, *MinEZK* Ministerie van Economische Zaken en Klimaat (Ministry of Economic Affairs and Climate), *MinOCW* Ministerie van Onderwijs, Cultuur en Wetenschap (Ministry of Education, Culture and Science), *NWO-TTW* Toegepaste en Technische Wetenschappen (Dutch Research Council—Applied and Technical Sciences), *WCN* Werkgroep Cardiologische centra Nederland, *ZonMw* Netherlands Organisation for Health research and Development

To guide the collaboration between partners, the DCVA is organised in six pillars (Research Policy, Valorisation, Implementation, Talent, Data Infrastructure, and Public Affairs and Communication). At the time of the analysis, the DCVA supported 32 consortia (see the Electronic Supplementary Material (ESM, Tab S1)), where collaborations between multiple research institutes, public and private organisations create a national network around specific cardiovascular themes. Some of these consortia were initiated by partners within the DCVA before the establishment of the DCVA and are strengthened by its connecting role and the financial and strategic support for the valorisation of results. The DCVA partners, Dutch Heart Foundation (DHF), Dutch Research Council (NWO), and Netherlands Organisation for Health Research and Development (ZonMw) are the main funders of these consortia (Fig. 1), with the largest contribution from the DHF. The agendas of the DCVA partners are a leading factor in selecting projects to be supported by the DCVA.

To evaluate the progress towards impact on cardiovascular health, the DCVA defined a Roadmap Health Technology Assessment (HTA) in collaboration with the section Health Technology & Services Research (HTSR) from the University of Twente. A DCVA HTA Steering committee was established including 13 experts from HTSR, DCVA and its partners (see the Electronic Supplementary Material (ESM, Tab S2)). HTA is a multidisciplinary process that uses quantitative methods to determine the value of a health technology at different points in its lifecycle [9]. Likewise, HTA can be used to assess the potential benefits of technological and non-technological innovations developed in DCVA consortia. More specifically, guidance on how to conduct HTA and economic evaluations which measure the costs and effectiveness of interventions in cardiovascular disease has recently been published [10]. The aim of this paper is to describe the activities of the partners within the DCVA, how these relate to the goal of reducing the CVD burden, and to explore in a quantitative analysis the likely health benefits generated by the DCVA consortia.

## Methods

An analysis of the activities and impact of the DCVA pillars Valorisation, Implementation, Infrastructure, and Talent was conducted (see ESM). Using a developed checklist, a qualitative assessment of the potential health economic impact of the 32 consortia was conducted, followed by a quantitative assessment of the health economic impact as measured by quality-adjusted life years (QALYs) and costs. Finally, an exploratory analysis of the contribution of the consortia to the goal of reducing the CVD burden (see ESM, Tab S3).

### Part A) Qualitative assessment of consortia

The NWO Impact Plan Approach describes the connection between research output and productive interactions/knowledge utilisation, and how these would ultimately create societal impact [11]. Using these definitions, a checklist was developed to evaluate impact, in consultation with the DCVA HTA Steering Committee and invited principal investigators of the consortia. The checklist was developed to capture the expected health and economic impact of the wide range of consortia. Care was taken to ensure that all facets of impact were captured, such as reductions in morbidity, improvements in patient experience, and healthcare workforce capacity.

The checklist is shown in the ESM Tab S4. Using the originally submitted project proposals from the consortia, the checklist was completed by HTSR, and reviewed by the DCVA HTA Steering committee on behalf of the individual consortia.

### Part B) Quantitative assessment of consortia in terms of health benefits from QALYs

The next step was a quantitative assessment of a snapshot of DCVA consortia (*n* = 4) as expressed in QALYs and costs. Based on the results of Question 2 ‘Pathway’ and Question 3 ‘Clinical Area’ from the qualitative assessment in Part A (Tab S1), four consortia were selected for the in-depth quantitative impact analysis. These consortia were chosen in consultation with the DCVA HTA Steering Committee to represent a breadth of projects, with Check@Home, LoDoCo2, IMPRESS, CONTRAST 2.0, representing Screening (CVD, chronic kidney disease (CKD), Diabetes), Treatment (coronary artery disease (CAD)), Diagnosis (CAD-women), and Treatment (Stroke), respectively (refer to ESM Tab S5).

The analysis was performed from a healthcare perspective. The incident patient numbers (prevalent for screening) were based on the Dutch population and extrapolated, based on a historical trend, to the year 2023. Costs were converted to Euros (€) [12] and inflated to 2023 Euros using the Dutch consumer price index [13]. A summary of the main steps taken to estimate the health economic impact of research in the consortia is shown in the Infobox. The key inputs and assumptions for the four studies are described in the ESM, Tables S6–S10.

#### Infobox Steps to measure the health economic impact of a consortium

1. Estimate the number of patients using prevalence (if applicable), screened population (if applicable) and/or incidence (if applicable). If applicable, adjust for the percentage of patients with a certain disease/condition, and the percentage of patients eligible for inclusion.
2. Estimate health outcomes with usual care vs. intervention, including estimations of:
  - The potential effect on the primary outcome measure that is studied;
  - The potential effect on resource utilisation and/or translation into health events (e.g. MACE events); and
  - The potential effect on health outcome expressed in QALYs (if applicable).
3. Use the findings from step 2b to estimate the costs of usual care vs. the cost of the intervention.
4. Estimate the additional costs and health benefits adjusted for the percentage of implementation of the intervention.

*MACE* myocardial infarction, stroke, or cardiovascular death, with or without coronary revascularisation, *QALYs* quality adjusted life year

### Part C) Estimation of CVD burden and contribution of the consortia to the goal

A crude exploratory analysis was also performed, to extrapolate consortia impact and estimate how all DCVA consortia combined could contribute to reducing the CVD burden (see ESM).

## Results

Key results have been presented here and the ESM. The complete results of all analyses are available upon request.

A review of the activities of the Valorisation, Implementation, Infrastructure and Talent pillars identified some concrete examples of the DCVA activities and their impact. In terms of the Valorisation pillar, the support in providing initial funding to attract external funding can be measured as the total volume of initial investment and how this translates into attracting funding from other external sources. The multiplier measures the redirection of funds to a company, i.e. the total extra investment. An example in the Infrastructure pillar includes the running of the Hartenbank (Heart Tissue Bank). This is a central biobank which requests tissue from donated organs with a focus on conducting research on genetic diseases.

### Part A) Qualitative assessment of consortia

**Fig. 2:**
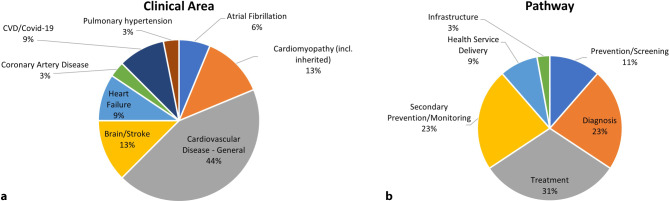
Summary of the clinical area [left] and pathway [right] from the qualitative assessment of consortia. *CVD* cardiovascular disease

The results of the ‘Clinical Area’ and ‘Pathway’ questions within the 32 reviewed consortia are presented in Fig. 2. The most common clinical area was CVD—General (44%) followed by cardiomyopathy (13%) and brain/stroke (13%). Most of the consortia focused on treatment (31%), followed by secondary prevention/monitoring (23%) and diagnosis (23%). Importantly, 31 consortia (97%) aim to reduce CVD-related morbidity, and 21 (66%) aim to (also) reduce CVD mortality. The additional steps to translate the ‘Outcomes’ of consortia into ‘Impact’ are presented in Tab. 1. Key results from the checklist are presented in the ESM (Tab S11–Tab S13).

**Table 1:** Additional steps needed to achieve the potential impact from the qualitative assessment of consortia

Additional Step	*N*	%
Uptake/implementation of clinical guidelines	31	97
Uptake of new diagnostic/therapeutic by clinicians	22	69
Translation of research towards clinical application	16	50
Intellectual Property rights	14	44
Reimbursement of diagnostic/therapeutic	13	41
Collaborate with industry to facilitate market access	12	38
Acceptance/adherence of new technology by patient	11	34
Acceptance of new technology by family/caregivers	9	28
Public/Private partnerships, e.g. service partners/affiliated businesses	8	25
Training of healthcare providers to use diagnostic or therapeutic	7	22
Changes to legislation and regulation	6	19
Reimbursement of clinician time/consultation	5	16
Future research projects using new database/infrastructure	3	9
Infrastructure adaptation with third-party	1	3
Development of financing structure, e.g. membership fee from patients	1	3
*Sum—additional step*	*159*	*497*^*†*^

† More than one answer is possible, therefore, the sum is greater than 100%

### Part B) Quantitative assessment of consortia in terms of health benefit from QALYs

In total, the estimated QALYs (cumulative to 2030) of the four projects was 8,494 [Check@Home 1,694; LoDoCo2: 362; IMPRESS: 3,655, CONTRAST 2.0: 2,783] with potential for associated cost savings in Check@Home and LoDoCo2. Detailed results are further described in the ESM (Tab S14–Tab S17).

### Part C) Estimation of CVD burden and contribution of consortia to the goal

The results of the exploratory analysis are reported in the ESM (Tab S18–Tab S21). The main assumption was that the extrapolation of benefit from Part B was generalisable to all 32 consortia. The extrapolated potential reduction in CVD burden was 78% (72 to 86% in sensitivity analysis).

## Discussion

This study shows that the DCVA consortia cover a wide range of clinical areas, with most consortia aiming to improve diagnostic strategies, including risk stratification, or to further personalize and improve diagnostic and treatment strategies. Within consortia, there is a clear view on the additional steps needed to achieve the intended impact. These key steps include adoption in clinical practice and securing reimbursement. For fundamental research, they involve translational activities to bring innovations into clinical application and the management of intellectual property rights. This indicates that the impacts of these consortia directly align with the DCVA ambition, with many consortia having additional impact related to patient satisfaction, access to care, improvements in healthcare workforce capacity and/or costs reduction.

### Strengths and limitations

A strength of this study is the comprehensive nature of the developed checklist. Future validation of the checklist against the reported results of the consortia can further strengthen the validity of the checklist. A highly simplified and pragmatic application of (early) HTA methods to quantify the potential health economic impact of consortia was adopted. This approach allowed the potential benefits of consortia to be quantified with a limited time, and without the need to wait years for the research to be completed and for their results to be implemented in clinical practice. Published articles using pragmatic early HTA approaches to rapidly evaluate a wide range of studies and innovations are lacking. Unfortunately, this prohibits comparing our results to similar impact analyses.

As mentioned in the Introduction, it is not possible to determine how much of the reduction in CVD burden achieved by the partners within the DCVA is attributable to the DCVA itself. Methods to quantitatively evaluate the value of scientific and policy collaboration are lacking, and the impact that the partners within the DCVA would have made without the DCVA is unknown. Still, learning how activities by the DCVA and partners contribute to the overall DCVA goal of reducing the CVD burden, and how to quantify the impact of research consortia, can support the DCVA and partners in their policymaking and decisions towards 2030.

There are several limitations regarding the exploratory quantitative assessment of the impact of the consortia (Part C). Therefore, in this exploratory analysis, a major assumption was that the mix of the four selected studies was a representative sample of all 32 consortia. This may lead to an over- or under-estimation of the expected reduction in CVD burden. In addition, this analysis did not include the potential benefits related to improvements in equity and healthcare service capacity. Also, while simplified early HTA methods work well for treatment interventions, they do require additional assumptions when evaluating screening and diagnostic innovations, given the complex and indirect relationship between diagnosis and health outcomes. Finally, many, if not all, consortia are expected to produce health benefits in the years or decades beyond 2030, while these benefits are not included in the current analyses.

### Future work—from retrospective to prospective HTA

Results indicated that additional steps are needed to translate the ‘Outcomes’ of consortia into ‘Impact’ for society. This highlights the importance of non-research activities, including implementation, to support the research consortia in actually creating impact. Although retrospective impact analyses can help determine if the DCVA is on track and aligned with its ambition, prospective impact analyses allow DCVA partners to further focus the efforts and resources on activities that are most likely to contribute substantially to the shared ambition. Prospective impact analyses can inform research prioritization decisions made during grant rounds, when ‘expected health benefits’ are added as an additional evaluation criterion. Such a criterion can also be extended to provide insight into improvements in other health-related outcomes, such as the number of years lived in good health and the reduction in health inequality, as formulated in goals of the Dutch Ministry of Health [8].

## Conclusion

The research activities of the DCVA partners cover a wide range of clinical areas with a focus on improving diagnostic strategies, including risk stratification, and the personalization of diagnostic and treatment strategies. Given the DCVA’s objective to reduce the CVD burden by 25% by 2030, our study indicates that the current DCVA activities and DCVA consortia contribute substantially towards reaching this goal. Reaching this goal also requires overcoming the many barriers and time delays in translating science into practice [14, 15]. Therefore, in addition to research, the support and promotion of valorisation, implementation, data infrastructure, public affairs, and talent development are crucial in achieving this goal. This analysis underscores the benefits of collaboration between DCVA partners in the cardiovascular field, which will continue in the future.

## Supplementary Information

Additional details, methods and results of the analysis.
