# Supplementary material for: Making a difference: describing and evaluating the impact of the Dutch CardioVascular Alliance
Source: Neth Heart J. 2025 Aug 14;33(10):307–12. doi: 10.1007/s12471-025-01975-y (PMC12454727; doi:10.1007/s12471-025-01975-y)
Supplement: Supplementary file 1 — Additional details, methods and results of the analysis. [file 12471_2025_1975_MOESM1_ESM.docx]

# Electronic Supplementary Material

## DCVA Consortia

Table 1: List of consortia reviewed

| 1. ARENA-PRIME | 17. Genius-II |
| --- | --- |
| 2. ARMCoach4Stroke | 18. HBCx |
| 3. Benefit | 19. Heart4Data |
| 4. CAPACITY | 20. HEROES |
| 5. CARRIER | 21. Holland Hybrid Heart |
| 6. CARE-ON | 22. **IMPRESS** |
| 7**. Check@Home** | 23. In Control II |
| 8. **CONTRAST 2.0** | 24. **LoDoCo2** |
| 9. COVID@Heart | 25. MyDigiTwin |
| 10. CUSTOM-AF | 26. OUTREACH |
| 11. Decision | 27. PERFECT FIT |
| 12. DEFENCE | 28. Phaedra-Impact |
| 13. Double Dose | 29. Predict 2 |
| 14. eCG family clinic | 30. RACE-9 |
| 15. ECG Project UMCU | 31. RECONNEXT |
| 16. FORSEE | 32. STRAP |

Note: Bolded consortia were included in the in-depth quantitative analysis

## DCVA Health Technology Assessment Steering Committee

Table 2: List of DCVA HTA Steering committee

| **Name** | **Affiliation** | **Consortia** |
| --- | --- | --- |
| Sopany Saing | Department Health Technology & Services Research, Technical Medical Centre, Faculty of Behavioural, Management and Social Sciences, University of Twente | N/A |
| Astrid Schut | Dutch Cardiovascular Alliance (DCVA)  Werkgroep Cardiologische centra Nederland (WCN), Utrecht | LoDoCo2 |
| Thea van Asselt | Department of Epidemiology & Department of Health Sciences, University of Groningen, University Medical Center Groningen | DECISION |
| Margien Boels | Dutch Cardiovascular Alliance (DCVA) | N/A |
| Eric Boersma | Department of Cardiology, University of Erasmus, University Medical Center Erasmus | IMPRESS |
| Wiek van Gilst | Dutch Cardiovascular Alliance (DCVA) | N/A |
| Clara van Ofwegen-Hanekamp | Nederlandse Vereniging Voor Cardiologie (NVVC) and Diakonessenhuis Utrecht | N/A |
| Jolien Roos‑Hesselink | Dutch CardioVascular Alliance (DCVA), Utrecht  Erasmus Medical Centre Rotterdam, Rotterdam | N/A |
| Frans Rutten | Julius Centre, University of Utrecht, University Medical Center Utrecht | N/A |
| Naomi Tramper | Dutch Cardiovascular Alliance (DCVA) | N/A |
| Robert Willemsen | Maastricht University, Maastricht, The Netherlands | N/A |
| Michelle Kip | Department Health Technology & Services Research, Technical Medical Centre, Faculty of Behavioural, Management and Social Sciences, University of Twente | N/A |
| Hendrik Koffijberg | Department Health Technology & Services Research, Technical Medical Centre, Faculty of Behavioural, Management and Social Sciences, University of Twente | Check@Home |

## A review of the activities of the Valorisation, Implementation, Infrastructure and Talent pillars that can contribute to the reduction in CVD burden.

A brief review of four of the pillars (Valorisation, Implementation, Data Infrastructure and Talent) was conducted to obtain insight into how the activities within these pillars contribute to the reduction of CVD burden. Due to time constraints the Public Affairs and Communication pillar was not reviewed and hence the potential impact of the activities from this pillar have not been described.

The interview broadly covered the following questions:

- How would you measure impact?
- How do you support the other pillars, especially the consortia?
- How might the pillar activities contribute to improving equity in healthcare (if applicable)?
- In what environment would the pillar function best? What needs to change in the system, either policy or healthcare workforce or administration for this pillar to work most efficiently?

There is a clear link between the activities performed, and support offered, by these pillars and the research executed by the consortia. For example, pillars can contribute to CVD burden reduction by either improving access to healthcare (Implementation) or ensuring that the conduct of clinical and translational work is more efficient (the Hartenbank from the Data Infrastructure pillar). For example, measuring impact in the Valorisation pillar could involve quantifying the increase in requests for intellectual property rights or the multiplier effect of attracting funding to the DCVA consortia.

Table 3: Summary of the review of the activities of the Valorisation, Implementation, Infrastructure and Talent pillars

| Function Overview | How to measure impact?ꝉ | Ideal situation |
| --- | --- | --- |
| Valorisation pillar | | |
| Impact officers assist the scientists from the consortia in their efforts to valorise their output. The impact officers are available for all researchers connected to the DCVA. They actively scout in existing consortia for potential spin off ideas. The role of the impact officer is to help conduct a needs assessment and support the soft development of a product, through:   - Providing education about valorisation. - Providing networking opportunities for scientists. - Providing complimentary consultation with a patent attorney. - Help building relationships with industry, (e.g. with medical device companies such as Phillips). - Offer funding/investment in the form of First Funds and Vouchers to support the research consortia in attracting further external funding. | - Quantify an increase in cardiac intellectual property requests, as intellectual property rights are often the basis for founding successful companies. - Estimate the timeline for events to get a product to market, potentially from the moment the company is founded until market access. Compare this time with and without the existence of the Valorisation pillar. - Estimate the Valley of Death / failure rate with and without the existence of the Valorisation pillar. - Multiplier Concept – measure the total volume of initial investment and how this translates to how the pillar is attracting funding from other external sources. The multiplier measures the re-direction of funds to a company, i.e. the total extra investment. For instance, ARENA PRIME (via Phlox), CONTRAST (via TargED) and River Biomedics (by way of Robert Passier, UT) obtained a total FIRST Fund of €3.4 million, this led to a subsequent external investment of €50 million, i.e. a 15.1 times multiplier | - Earlier engagement of researchers with an impact officer. Ideally, before a research grant is written so that a needs assessment is developed with input from relevant stakeholders. The impact officer can promote the development of these productive interactions to patients, industries, carers, policymakers and guideline committees. |
| Implementation pillarꝉ | | |
| - Provide support to implement new solutions in daily practice at hospitals and other health care providers with the goal to enable ‘fast tracks from lab to patient’. - Discuss innovations/policies/programmes with proven benefits that warrant implementation. Here, the scope is very broad, and discussions take place both during the research and post-research phase. The main aim is to determine how doctors (and other healthcare professionals) are going to use the innovation, and how to get this innovation reflected in the relevant guidelines. - Investigate the shortcomings and barriers to implementation of digital health innovations. The pillar can also provide training, e.g. e-Health systems training to GPs and nurses. | - This pillar can have an impact on equity and delivery of health care. There is an overarching goal of preventing a shortage of healthcare, both in terms of access and in terms of healthcare workforce capacity. There is a particular focus on improving access to healthcare for those of a lower socioeconomic status. - Improve health and digital literacy and make digital healthcare more accessible. Be aware of barriers to implementation including but not limited to skepticism either from patient or from the workforce. - Potential to influence transformation of care from hospital to GPs or home. Similarly, for patients treated in hospitals to be cared for by nurses rather than cardiologists or other medical specialists. The pillar can be aware of barriers to shifting burden of care in practice and ensure that patient access to care is optimized. | - Improve involvement of patients in their own care. - Improve representation with partner organization. - Improve collaboration with reimbursement agencies as reimbursement is crucial for successful implementation. |
| Data infrastructure pillar | | |
| - Assist in funding applications for researchers to share their data and support open science principles including Findable, Accessible, Interoperable, and Reusable (FAIR). - Development of sustainable research infrastructure, i.e. Heart4Data quality registries to maximize the potential of registry-based research using observational data. - Running of the Hartenbank (Heart Tissue Bank), this is a central biobank which requests tissue from donated organs with a focus on conducting research in genetic diseases. | - Measure utilization of the service (a catalogue of all data), i.e to measure how much data or samples are used (and reused) by researchers. Can also measure the number of data request from outside the DCVA (i.e. external groups). - Measure time not spent on collecting and cleaning data due to the service. For instance, PhD students can spend more time on data analysis and not data cleaning, therefore the service could potentially automate tasks. - Quantify the assistance of the service in obtaining research income, i.e. due to be able to list existing data availability in grant proposals. | - There is optimal governance and understanding of the value of a robust and sustainable data infrastructure. A more complete data linkage including cost data, primary care usage and mortality data is required for researchers. - A robust auditing system to ensure greater harmonization of data collection across all hospitals. - A more national approach to infrastructure (as is the case in oncology) and a less fragmented system as it is currently. Greater consistency with privacy and opt-in-and-opt out. |
| Talent pillar | | |
| Identify, train and support the talents of the cardiovascular community at large and create the next generation of leaders in the field. Host regular training events and the DCVA NLHI Translational Cardiovascular Research Meeting. Most persons work within research, clinical or academia with career opportunities including government, industry (SMEs in Biotech), Elsevier (publishing) and Patent Officer.  There are 3 supported talent groups broadly distinguished by career stage:   - Young@Heart –consisting of a group between 20 to 30 researchers who are still completing a PhD and are working towards a post-doctoral position. The group offers networking opportunities and training to become better researchers and better able to fund their own research. Researchers are also exposed to roles outside of academia, incl. entrepreneurial roles. - Steer-Your Career – consisting of a group of around 15 researchers who have either recently graduated or are a senior PhD student. The training program provides tailored career advice and training on how to write grant proposals, business canvas training and includes a valorisation component. Consists of an intense training program over 6 days a year. - Leadership Program – consists of 15 mid-career researchers over 2-years of training. Professional training for team interaction and leadership. Learn how to deal with complex issues e.g. lobbying and how to create a consortium. | - The development of spin-off/start-up companies from the DCVA and how this generates employability. Research in DCVA consortia have led to the creation of several more recent Dutch spin-off companies such as Phlox Therapeutics, River Biomedics and Summa Biotech where in total ~ 20 employees now are employed. Predating the DCVA, the CVON-initiated Dutch mid-sized Biotech company Lead Pharma Medicine now employs 30 personnel. Overall job creation both within and outside of research, clinical and academic. - Measure the number of early-or mid-career researchers who move into senior leadership positions. | - Intense training on grant writing would be available for all talent groups, grant writing support is currently only available at for researchers at the mid-career level. - Provision of more coordinated support for training. For example, a dedicated person to continuously train for grant support that works across all different disciplines (multidisciplinary) and help to better facilitate the mobilization of funding e.g. ZonMW. - Greater reach of the talent network including, more engineers from Technical Universities as currently the focus is on researchers in biomedical/medical experts; or greater representation from other disciplines, e.g radiology or nursing. |

Note: ^ꝉ^ The implementation pillar question of ‘How to measure impact?’ was reframed as a question of ‘What is the impact on equity?’

Abbreviations: DCVA = Dutch Cardiovascular Alliance, GPs = general practitioners, SME= small medium enterprises, UT = University of Twente, ZonMw = Netherlands Organisation for Health research and Development

## Impact checklist for DCVA consortia

Table 4: Impact checklist for dcva consortia

| Criteria | *Possible response(s)* |
| --- | --- |
| **1.Title** | *Short and long title of the consortium* |
| **2.Pathway** | Question: Specify the pathway to which your project belongs. |
|  | - Prevention/Screening - Diagnosis - Treatment - Secondary Prevention/Monitoring - Health Service Delivery - Infrastructure |
| **3.Clinical area** | Question: Specify the clinical area to which your project belongs. |
|  | - Atrial Fibrillation - Cardiomyopathy (incl. inherited) - Cardiovascular Disease - General - Brain/Stroke - Heart Failure - Coronary Artery Disease - CVD/Covid-19 - Pulmonary hypertension |
| **4.Output** | Question: List the key output that the project will/has generate(d)?  Open text |
| **5.Outcomes** | Question: How will this output of this programme/research lead to improved outcomes, either directly or in the future? [Tick all that apply] |
|  | - Improve diagnosis in patients - Changes to infrastructure and methodology - Identify new biomarkers/pathophysiological mechanism - Develop new therapeutics - Provide personalised medicine - Improve risk stratification in patients - Prevention of flow-on consequence of CVD - Improve continuity between care levels (secondary and primary) |
| **6.Additional Steps** | Question: Which requirements are needed before this impact is reached? [Tick all that apply] |
|  | - Infrastructure adaptation with third-party - Intellectual Property rights - Changes to legislation and regulation - Translation of research towards clinical application - Uptake of new diagnostic/therapeutic by clinicians - Training of healthcare providers to use diagnostic or therapeutic - Development of financing structure, e.g. membership fee from patients - Collaborate with industry to facilitate market access - Public/Private partnerships, e.g. service partners/affiliated businesses - Reimbursement of diagnostic/therapeutic - Reimbursement of clinician time/consultation - Future research projects using new database/infrastructure - Acceptance/adherence of new technology by patient - Acceptance of new technology by family/caregivers |
| **7.Impact** | Question 7a: How could the outcomes in the previous questions contribute to lower CVD burden (impact)?  [Tick all that apply].  Question 7b: How could the outcomes in the previous questions contribute to lower CVD burden (impact)?  [Tick the option that most resonates with the expected impact of the project]. |
|  | - Improvements in mortality (e.g. reduction in deaths due to heart failure) - Improvements in morbidity (e.g. reduction in complications and reliance on surgery) - Reduction in incidence of CVD - Appropriate care in patients groups with previously unmet need (e.g. equity in terms of gender or rare-disease) - Improvements in patient experience/patient satisfaction (e.g. comfort) - Improvements in patients access to care (e.g. convenience) - Improvements in healthcare workforce capacity - Reduction in costs to the healthcare system - Reduction in productivity losses to the patient - Reduction in research costs |

Abbreviations: CVD = cardiovascular disease, DCVA = Dutch Cardiovascular Alliance.

## Details of the four case-studies for the quantitative assessment of impact

Table 5: Case-studies for the quantitative assessment of impact

| Title | Pathway | Clinical Area | Intervention |
| --- | --- | --- | --- |
| **Check@Home** | Screening | CVD | At-home screening every two years, in men and women aged 50 to 75 years to detect cardiovascular disease using atrial fibrillation CE-certified Happitech app, photoplethysmographic signals; questionnaire Reviving Early Diagnosis of CardioVascular Disease (RED-CVD) for coronary artery disease and heart failure |
|  |  | CKD | At-home screening every two years, in men and women aged 50 to 75 years to detect chronic kidney disease using albuminuria test, PeeSpot + albuminuria test |
|  |  | Diabetes | At-home screening every two years, in men and women aged 50 to 75 years to detect Type 2 diabetes |
| **LoDoCo2** | Treatment | CAD | Colchicine tablets (0.5 mg) for the treatment of CAD |
| **IMPRESS** | Diagnosis | CAD - Women | Dutch consortium to improve the care of women with CVD - develop National Knowledge Platform |
|  |  |  | 1) Development and validation of a sex-specific decision support tool for general practitioners in women <60 years presenting with symptoms of potential cardiac origin using classical regression methods and machine learning techniques |
|  |  |  | 2a) A decision support tool to rule out MVA/VSA in women with persistent signs and symptoms of ischemia, using invasive coronary angiography (CAG) as gold standard. Performing systematic interventional diagnostic procedures (IDPs) defined as the intracoronary acetylcholine testing for the detection of coronary spasm as well as coronary flow reserve and microvascular resistance assessment in response to adenosine using a dedicated wire. |
|  |  |  | 2b) Test which non-invasive tools (CT perfusion, cardiac MRI and high-resolution body surface mapping) show high correlations with MVA/VSA after IDPs |
|  |  |  | 2c) Treatment of MVA with calcium channel blocker (CCB) diltiazem 120-360 mg/day in the EDIT-CMD (Efficacy of DilTiazem to improve Coronary Microvascular Dysfunction), a placebo-controlled trial. Diltiazem previously prescribed for treatment of VSA. |
|  |  |  | 3) Unravel if trained immunity and autoimmune responses predispose to an enhanced risk of plaque erosion (accelerated atherosclerosis) in younger women (with preeclampsia)‡ |
| **CONTRAST 2.0^†^** | Treatment | Stroke | An integrated research program that develops and evaluates new treatment strategies for acute ischemic stroke, intracranial hemorrhage (ICH) and subarachnoid hemorrhage (SAH) in preclinical studies, and a series of complementary RCTs and registries**^†^**. |
|  |  |  | 1) Trial 1 [MR CLEAN MeVO] - endovascular treatment (EVT) in medium vessel occlusion (MeVO). |
|  |  |  | 2) Trial 2 [MR CLEAN CASES] - immediate carotid stenting for steno-occlusive disease versus delayed carotid endarterectomy. |
|  |  |  | 3) Trial 3 [DIST]- minimally invasive endoscopy-guided surgery for cerebellar intracerebral hemorrhage (ICH). |

**^†^**For the purposes of this analysis the three observational studies were not evaluated. ‡The sub-study was not evaluated due to limited data.

Abbreviations: CT = computerized tomography, DIST = Dutch Intracerebral hemorrhage Surgery Trial, MRI = magnetic resonance imaging, MVA = microvascular angina, RCT = randomized controlled trial, VSA = vasospastic angina

## Key inputs and assumptions for CHECK@HOME

Table 6*:* parameters and key assumptions - Check@Home

| Description | Value (Range) | Assumption/Formula | Ref. |
| --- | --- | --- | --- |
| Estimation of treatment effect in CVD - AF | | | |
| Probability of NOAC compared to VKA | 0.50 | p_NOACvVKA Assume treatment with NOACS and VKAs are equally likely. | (1) |
| Probability of minor IS event under treatment | 0.0059 (0.0054-0.065) | Yearly probability of minor IS converted from 3-month event probability of minor IS weighted by treatment. p_IS_minor_treatment = [(0.0014*p_NOACvVKA)+(0.00157*(1-p_NOACvVKA))]*4  Yearly probability of minor IS converted from 3-month event probability of minor IS weighted by treatment. p_IS_minor_treatment_upper = [(0.00156*p_NOACvVKA)+(0.00171*(1-p_NOACvVKA))]*4  Yearly probability of minor IS converted from 3-month event probability of minor IS weighted by treatment. p_IS_minor_treatment_lower = [(0.00126*p_NOACvVKA)+(0.00142*(1-p_NOACvVKA))]*4 |  |
| Probability of major IS event under treatment | 0.0037 (0.0034-0.0041) | Yearly probability of major IS converted from 3-month event probability of major IS. p_IS_major_treatment = [(0.00088*p_NOACvVKA)+(0.00099*(1-p_NOACvVKA))]*4  Yearly probability of major IS converted from 3-month event probability of major IS. p_IS_major_treatment_upper = [(0.00097*p_NOACvVKA)+(0.00108*(1-p_NOACvVKA))]*4  Yearly probability of major IS converted from 3-month event probability of major IS. p_IS_major_treatment_lower = [(0.00079*p_NOACvVKA)+(0.00089*(1-p_NOACvVKA))]*4 |  |
| Probability of fatal IS event under treatment | 0.0009 (0.0008-0.0009) | Yearly probability of fatal IS converted from 3-month event probability of fatal IS. p_IS_fatal_treatment = [(0.0002*p_NOACvVKA)+(0.00023*(1-p_NOACvVKA))]*4  Yearly probability of fatal IS converted from 3-month event probability of fatal IS. p_IS_fatal_treatment_upper = [(0.00022*p_NOACvVKA)+(0.00025*(1-p_NOACvVKA))]*4  Yearly probability of fatal IS converted from 3-month event probability of fatal IS. p_IS_fatal_treatment_lower = [(0.00018*p_NOACvVKA)+(0.0002*(1-p_NOACvVKA))]*4 |  |
| Probability that the IS event is either minor, major or fatal if treated | 0.56:0.35:0.08 | p_IS_total_treatment Probability that the IS event is either minor, major or fatal if treated to estimate distribution of IS event. |  |
| Probability of minor IS event with no treatment | 0.015 | Yearly probability of minor IS converted from 3-month event probability of minor IS. p_IS_minor_notreatment = 0.00378*4 |  |
| Probability of major IS event with treatment | 0.014 | Yearly probability of major IS converted from -month event probability of major IS. p_IS_major_notreatment  = 0.00345*4 |  |
| Probability of fatal IS event with treatment | 0.008 | Yearly probability of fatal IS converted from 3-month event probability of fatal IS. p_IS_fatal_notreatment.  = 0.0021*4 |  |
| Probability that the IS event is either minor, major or fatal if treated | 0.41:0.37:0.22 | p_IS_total_notreatment. Probability that the IS event is either minor, major or fatal if untreated to estimate distribution of IS event. | Calculated. |
| Effect of intervention | 0.011 (0.009-0.0012) | 1-year probability of IS event due to AF as defined by minor, major or fatal IS after detection and on treatment  = (p_IS_minor_treatment+p_IS_major_treatment+p_IS_fatal_treatment)  = (p_IS_minor_treatment_lower+p_IS_major_treatment_lower+p_IS_fatal_treatment_lower)  = (p_IS_minor_treatment_upper+p_IS_major_treatment_upper+p_IS_fatal_treatment_upper) | Calculated |
| Effect of usual care | 0.037 | 1-year probability of IS event due to AF as defined by minor, major or fatal IS without treatment as undetected  = (p_IS_minor_notreatment+p_IS_major_notreatment+p_IS_fatal_notreatment) | Calculated |
| Estimation of treatment effect in CVD - HF | | | |
| Effect of intervention | 0.10 (0.052, 0.148) | p_1yr_HF_detected_CHF. 1-year probability of HF event defined as risk of HF hospitalisation, IHD, stroke, and all-cause death adjusted for HF with HF stage B | (2) |
| hr_hf_treatment | 0.44 (0.26-0.66) | Hazard ratio treatment for heart failure with ACEI+BB+ARB, all-cause mortality, network meta-analysis | (3) |
| p_HF_rr_treatment | 0.54 | Probability of HF event based on relative reduction due to treatment of CHF = 1-(hr_hf_treatment/(1-p_1yr_HF_detected_CHF*hr_hf_treatment)) | Calculated |
| Effect of usual care | 0.217 | 1-year probability of HF event with undetected CHF p_1yr_HF_undetected_CHF = p_1yr_HF_detected_CHF/(1-p_HF_rr_treatment) | Calculated |
| Estimation of treatment effect in CKD | | | |
| Effect of intervention | 0.041 (0.032-0.049) | 1-year probability of KF with detected CKD = p_1yr_KF_detected_CKD (p_1yr_KF_detected_CKD_upper - p_1yr_KF_detected_CKD_lower) | (4) and -/+ 20% |
| or_KF_treatment | 0.61 (0.47-0.79) | Odds ratio of KF reduction due to treatment with ACEI and ARBs compared to placebo | (5) |
| p_KF_rr_treatment | 0.37 | Probability of KF based on relative reduction due to treatment of CKD = 1-or_KF_treatment/(1-p_1yr_KF_detected_CKD*or_KF_treatment) | Calculated |
| Effect of usual care | 0.066 | 1-year probability of KF with undetected CKD = p_1yr_KF_detected_CKD/(1-p_KF_rr_treatment) | Calculated |
| Event estimation due to screening or no screening | | | |
| f_medication_adherence_AF  f_medication_adherence_CHD  f_medication_adherence_CKD  f_medication_adherence_T2D_HF | 1.1 | An adjustment for adherence to drug treatment in the Check@Home group. Despite a diagnosis from the screening arm, those in the Intervention arm do not always adhere to their medication/treatment and so the benefit of disease reduction is not reached in 10% of the population | Assumption |
| Detection of HF in T2D | 5.38 (4.23-6.76) | Incidence rate of heart failure per 1000 person-years, age 55-64 with diabetes = ir_HF_person_years_diabetes (ir_HF_person_years_diabetes_upper- ir_HF_person_years_diabetes_lower) | (6) |
| p_effectiveness_HF_Checkathome | 0.46 | Effectiveness of Check@Home program for HF screening  =p_1yr_HF_detected_CHF/p_1yr_HF_undetected_CHF | Calculated |
| Utility values | | | |
| Proportion enrolled in Stroke Service vs. UC in EDISSE† | 0.45 | p_ss_uc_EDISSE = 151/(151+187) | (7) |
| Minor IS (mRS 1-2) from AF, acute | 0.620 | u_IS_AF_minor_acute = 0.6245*p_ss_uc_EDISSE+0.6163*(1- p_ss_uc_EDISSE) | Calc. from (7) |
| Minor IS (mRS 1-2) from AF, post | 0.725 | u_IS_AF_minor_post = 0.7726* p_ss_uc_EDISSE+0.6863*(1- p_ss_uc_EDISSE) |  |
| Proportion of mRS 4 out of mRS 4/5, acute, SS | 0.67 | p_mRS4_maj_acute_ss = 31/(31+15) |  |
| Proportion of mRS 4 out of mRS 4/5, acute, UC | 0.81 | p_mRS4_maj_acute_uc = 29/(29+7) |  |
| Major IS (mRS 3-5)^‡^ from AF, acute, SS | 0.056 | u_IS_AF_major_acute_ss = 0.1667*p_mRS4_maj_acute_ss+(-0.1739)*(1-p_mRS4_maj_acute_ss) |  |
| Major IS (mRS 3-5)^‡^ from AF, acute, UC | 0.153 | u_IS_AF_major_acute_uc = 0.2238*p_mRS4_maj_acute_uc+(-0.1413)*(1-p_mRS4_maj_acute_uc) |  |
| Major IS (mRS 3-5)^‡^ from AF, acute, weighted | 0.109 | u_IS_AF_major_acute = u_IS_AF_major_acute_ss* p_ss_uc_EDISSE +u_IS_AF_major_acute_uc*(1- p_ss_uc_EDISSE) |  |
| Proportion of mRS 4 out of mRS 4/5, post, SS | 0.88 | p_mRS4_maj_post_ss = 23/(23+3) |  |
| Proportion of mRS 4 out of mRS 4/5, post, UC | 0.74 | p_mRS4_maj_post_uc = 17/(17+6) |  |
| Major IS (mRS 3-5)^‡^ from AF, post, SS | 0.633 | u_IS_AF_major_post_ss = 0.6758*p_mRS4_maj_post_ss+0.303*(1-p_mRS4_maj_post_ss) |  |
| Major IS (mRS 3-5)^‡^ from AF, post, UC | 0.457 | u_IS_AF_major_post_uc = 0.5351*p_mRS4_maj_post_uc+0.2371*(1-p_mRS4_maj_post_uc) |  |
| Major IS (mRS 3-5)^‡^ from AF, weighted | 0.536 | u_IS_AF_major_post = u_IS_AF_major_post_ss* p_ss_uc_EDISSE +u_IS_AF_major_post_uc*(1- p_ss_uc_EDISSE) |  |
| Proportion of acute/discharge phase vs. post-phase | 0.5 | p_IS_acute_vs_post = 6 months out of 12 months |  |
| Utility, IS Minor | 0.672 | u_IS_minor_weighted = u_IS_AF_minor_acute*p_IS_acute_vs_post+u_IS_AF_minor_post*(1-p_IS_acute_vs_post) |  |
| Utility, IS Major | 0.323 | u_IS_major_weighted = u_IS_AF_major_acute*p_IS_acute_vs_post+u_IS_AF_major_post*(1-p_IS_acute_vs_post) |  |
| Utility,weighted UC | 0.40 | p_IS_minor_total_notreatment*u_IS_minor_weighted+p_IS_major_total_notreatment*u_IS_major_weighted+p_IS_fatal_notreatment+u_IS_fatal |  |
| Utility, weighted, Intervention | 0.48 | (p_IS_minor_total_treatment*u_IS_minor_weighted+p_IS_major_total_treatment*u_IS_major_weighted+p_IS_fatal_treatment+u_IS_fatal)+u_dec_IS_anticoag |  |
| HF hospitalisation within previous 30 to 90 days | -0.054 (-0.062 to -0.045) | u_dec_HF_hospitalisation | (8) |
| On the KTx waiting list, male, aged 50-59 years old | -0.058 (−0.093 to −0.023) | u_dec_waiting_list_kidney | (9) |
| Year of HF event for people with diabetes | -0.183 (-0.258 to -0.108) | u_dec_HF_diabetes | (10) |
| Cost | | | |
| Cost of minor IS acute phase, per 3 mths | €19,146 (2014)  €24,489 (2023) | c_IS_minor_acute_3mth | (1) |
| Cost of minor IS post phase, per 3 mths | €1,484 (2014)  €1,898 (2023) | c_IS_minor_post_3mth | (1) |
| Cost of major IS acute phase, per 3 mths | €44,138 (2014)  €56,456 (2023) | c_IS_major_acute_3mth | (1) |
| Cost of major IS post phase, per 3 mths | €3,958 (2014)  €5,063 (2023) | c_IS_major_post_3mth | (1) |
| Cost of fatal IS | €11,178 (2014)  €14,297 (2023) | c_IS_fatal | (1) |
| Cost of NOAC, per 3 mths | €235 (2014)  €301 (2023) | c_NOAC_3mth | (1) |
| Cost of VKA, per 3 mths | €105 (2014)  €134 (2023) | c_VKA_3mth | (1) |
| Cost of NOAC, per year | €1,202 (2023) | c_NOAC_year | (1) |
| Cost of VKA, per year | €537 (2023) | c_VKA_year | (1) |
| Mean cost of hospitalization for chronic heart failure | €7,060 (2017) €8,826 (2023) | c_HF_hospitalisation | (11) |
| Haemodialysis for KF, 2014 | €92,616 | c_haemodialysis | (12) |
| Cost of transplantation with a deceased donor kidney, 2014 | €99,450 [SD€36,036] | c_transplant_deceased_donor | (12) |
| Cost of transplantation with a living donor kidney, 2014 | €73,376 [SD= €38,666] | c_transplant_living_donor | (12) |
| Number of live kidney donor transplantations in 2022 | 516 | n_kidney_live | (13) |
| Number of deceased kidney donor transplantations in 2022 | 492 | n_kidney_deceased | (13) |
| Cost of transplantation, weighted for deceased and living donor kidney, 2014 | €86,103 | c_transplant=c_transplant_deceased_donor*(n_kidney_deceased/(n_kidney_live+n_kidney_deceased))+c_transplant_living_donor*(n_kidney_live/(n_kidney_deceased+n_kidney_live)) | Calculated |
| Dialysis patients in 2020 | 18,000 | n_dialysis | (14) |
| Percentage of kidney failure patients who receive a KTx | 0.056 | p_kidney_transplant=(n_kidney_live+n_kidney_deceased)/n_dialysis | Calculated |
| Weighted average cost of haemodialysis, KTx (living donor and deceased donor) | €92,251 (2014) €117,996 (2023) | c_KF=c_haemodialysis*(1-p_kidney_transplant)+(c_transplant*p_kidney_transplant) | Calculated |

Note: Treatment with NOACs include apixaban, dabigatran, or rivaroxaban and VKA is warfarin. 3-month yearly probabilities converted to yearly probabilities.

Note: ^†^The EDISSE study (Evaluation of Dutch Integrated Stroke Service Experiments) is a prospective non-randomised controlled cluster trial conducted in 2001(15). Assume that acute phase utility value collected at discharge and that post phase are the utility values collected at 6 months. ‡Although minor IS is considered mRS3 to mRS 5, the EDISSE study as reported in Table 3, Baeten et al. (2010).

Abbreviations: ACEI = angiotensin-converting enzyme inhibitors, AF = atrial fibrillation, ARB = angiotensin receptor blockers, BB = beta-blockers, CHF = congestive heart failure, CKD = chronic kidney disease, HF = heart failure, IHD = ischaemic heart disease, IS = ischaemic stroke, KF = kidney failure, KTx = kidney transplant, mRS = modified Rankin Scale, NOAC = non-vitamin K antagonist oral anticoagulants, SD = standard deviation, SS = stroke service, T2D=Type 2 diabetes, UC = usual care, VKA = vitamin K antagonists.

## Key inputs and assumptions for LoDoCo2

Potential treatment effect

The primary endpoint in the LoDoCo2 trial was the composite endpoint of cardiovascular death, spontaneous (nonprocedural) myocardial infarction, ischemic stroke, or ischemia-driven coronary revascularization. The odds ratio of having an event as defined in the primary endpoint by treatment with 0.5 mg colchicine vs. placebo equated to 0.69 (95% CI 0.57-0.83, p<0.001) (16). In this analysis, the probability of a MACE event in the first year which equates to 6.8% in the intervention group vs. 9.6% in the usual care group was utilized from Nidorf et al. (2020) (16).

Potential effect on health outcomes in terms of quality adjusted life years

A recent paper from the LoDoCo2 trial noted that the individual lifetime benefit from low-dose colchicine in chronic CAD was a median 2.0 MACE-free years gained(17). However, for the purposes of the current analysis, QALY estimates were preferred. Therefore, the estimated lifetime benefit of low-dose colchicine was 0.04 QALY per patient (18). In the sensitivity analysis a QALY range of 0.03 and 0.05 was applied. This was based on a Markov cohort state-transition model from the LoDoCo2 trial assuming lifetime horizon with patients starting at age 66 years (18). In the Markov model, patients were clinically stable for at least 6 months and would enter the acute-state (and be on treatment) for a 1-year cycle, and then transition to the post-state until death or a new acute event (myocardial infarction, stroke and coronary revascularization) occurred (18).

Table 7: risks and costs of event - LoDoCo2

| Event | Mean cost (2016) | Mean annual event risk† |
| --- | --- | --- |
| Myocardial infarction | €5,037 | 1.26 |
| Stroke | €19,030 | 0.66 |
| Resuscitated cardiac arrest | €28,636 | 0.10 |
| Revascularisation (weighted average PCI and CABG) | €6,944 | 2.23 |
| Post-event care for chronic heart failure | €6,569 | 0.49 |
| Weighted average (2016) | €40,474 |  |
| Weighted average (2023) | €51,295 |  |

† For 60 year old from Berkelmans et al. (2020)(19)

Abbreviations: CABG = coronary artery bypass graft, PCI = percutaneous coronary intervention

## Key inputs and assumptions for IMPRESS

Potential effect on health outcomes in terms of quality adjusted life years

The estimated survival in terms of additional life years for a 50 year old women diagnosed with heart disease was 7.9 years (5.8 years with heart disease and 2.1 years after heart attack)(20). The same study estimated that for a 60 year old women diagnosed with heart disease, she could expect to live an additional 7.4 years (5.3 years with heart disease and 2.1 years after heart attack)(20). The use of a sex-specific decision support tool for GPs was expected to result in 5% (assumption) of CVD diagnoses to occur at age 50 rather than age 60. In the sensitivity analysis, it was assumed that the chance of MI being missed by the GP was 4% and 6%, respectively. This results in on average 7.43 years gained in the intervention group compared to 7.40 years gained in the usual care group.

The CorMicA trial (n=151) compared stratified medical therapy ‘intervention’ with ‘usual care’ after an IDP. In the CorMicA trial, the mean quality of life as measured by the EQ-5D-5L after 6 months in the intervention group was 0.66 (SD=0.28) and 0.50 (SD=0.4) in the control group (21). This difference resulted in a statistically significant improvement at 6 months with mean difference in quality of life of 0.10 (95% CI 0.01-0.18, p=0.024) adjusted for baseline values. The adjusted mean difference of 0.10 utility value was relied on in the current analysis (21).

Table 8: parameters and key assumptions IMPRESS

| Description | Value | Assumption/Formula | Ref. |
| --- | --- | --- | --- |
| **Treatment effect** | | | |
| Major adverse cardiac and cerebrovascular events for ICA vs. no ICA | 2.6% | No difference | (21) |
| Major adverse cardiac and cerebrovascular events for ICA vs. no ICA | 2.6% |  |  |
| MACE events after phenotyping with invasive vs. non-invasive imaging | 3.7% | based on sample size calculation for non-inferiority trial | (22) |
| Successful Treatment with diltiazem in patients with MVA - UC | 29% | 8% worse performance in intervention | (23) |
| Successful Treatment with diltiazem in patients with MVA - Intervention | 21% |  |  |
| Epicardial Spasm - UC | 54% | 23% reduction (improvement from intervention) | (23) |
| Epicardial Spasm - Intervention | 32% |  |  |
| **Costs** | | | |
| Decision support tool for GP - UC | €0 |  | Assumption |
| Decision support tool for GP - Intervention | €20 |  |  |
| ICA | €1,378 (2011) €1,869 (2023) |  | (24) |
| CT vs. ICA - UC | €1,378 (2011) €1,869 (2023) |  |  |
| CT vs. ICA - Intervention | €180 (2011) €244 (2023) |  |  |
| Diltiazem, annual cost | €14 (2023) |  | (25) |
| 5-year costs in women, nonobstructive CAD | US$32,239 (2003) €43,858 (2023) | c_unobstructive |  |
| 5-year costs in women, obstructive 3-vessel CAD | US$53,398 (2003) €72,642 (2023) | c_obstructive | (26) |
| Proportion of symptomatic women with non-obstructive CAD | 0.63 | p_women_nonobstructive_CAD | (26) |
| Number of years of costs | 5 | n_years_cost | (27) |
| Weighted average annual healthcare costs | €10,901 (2023) | (c_unobstructive * p_women_nonobstructive_CAD + c_obstructive*(1-p_women_nonobstructive_CAD)) / n_years_cost | - |

Abbreviations: CAD = coronary artery disease, CT= computed tomography, GP = general practitioner, ICA = invasive coronary angiography, MACE = myocardial infarction, stroke, or cardiovascular death, with or without coronary revascularisation (PCI or CABG), MVA = microvascular angina, UC = usual care

## Key inputs and assumptions for CONTRAST 2.0

Table 9: Parameter – CONTRAST 2.0

| Description | Value | Ref. |
| --- | --- | --- |
| Proportion | | |
| Stroke due to large-vessel occlusion | 38% (35%-40%) | (28) |
| Stroke due to medium-vessel occlusion | 32% (24%-40%) | (28) |
| Intracerebral hemorrhage of all strokes | 15% | CONTRAST 2.0 Proposal |
| LVO patients with high-grade stenosis in the ipsilateral cervical carotid artery | 20% | (29) |
| Cost | | |
| Endovascular treatment | €9,925 (2021)  €11,430 (2023) | (30) |
| carotid endarterectomy (CEA) | €5,500 (2003)  €6,188 (2023 | (31) |
| carotid artery stenting (CAS) | €4,012 (2003)  €8,483 (2023) |  |
| Minimally invasive neurosurgery in ICH | €10,000 (2019)  €11,976 (2023) | (32) |

Abbreviations: ICH = intracerebral haemorrhage, LVO = large vessel occlusion

Table 10: proportion, cost and utility values by modified rankin scale - CONTRAST 2.0

| Description | | mRS 0 | mRS 1 | mRS 2 | mRS 3 | mRS 4 | mRS 5 | mRS 6 | Ref. |
| --- | --- | --- | --- | --- | --- | --- | --- | --- | --- |
| Proportion and Treatment effect | | | | | | | | | |
| EVT MeVO | Intervention | 0.030 | 0.090 | 0.210 | 0.180 | 0.220 | 0.060 | 0.210 | 1.67^ꝉ^ (33) |
|  | Usual care | 0.010 | 0.060 | 0.130 | 0.160 | 0.300 | 0.120 | 0.220 |  |
| CASES | Intervention | 0.179 | 0.223 | 0.167 | 0.144 | 0.123 | 0.050 | 0.114 | 1.15^ꝉ^  (29) |
|  | Usual care | 0.069 | 0.202 | 0.179 | 0.164 | 0.172 | 0.080 | 0.134 |  |
| DIST - ICH | Intervention | 0.022 | 0.129 | 0.189 | 0.212 | 0.158 | 0.106 | 0.183 | 1.49^ꝉ^ (32) |
|  | Usual care | 0.002 | 0.109 | 0.160 | 0.171 | 0.195 | 0.143 | 0.220 |  |
| Cost | | | | | | | | | |
| LVO | 2015 | €10,382 | €12,131 | €14,570 | €21,629 | €24,978 | €27,442 | €11,242 | (34) |
| LVO | 2023 | €13,200 | €15,424 | €18,525 | €27,499 | €31,757 | €34,890 | €14,293 |  |
| ICH | 2019 | €16,012 | €13,026 | €18,447 | €33,952 | €34,237 | €72,303 | €5,612 | (32) |
| ICH | 2023 | €19,176 | €15,600 | €22,093 | €40,662 | €41,003 | €86,592 | €6,721 |  |
| Utility | | | | | | | | | |
| Mean EQ-5D | mean LVO | 0.95 | 0.93 | 0.83 | 0.62 | 0.42 | 0.11 | 0 | (33) |
|  | LVO – lower | 0.89 | 0.89 | 0.79 | 0.56 | 0.37 | 0.03 | 0 |  |
|  | LVO - upper | 1 | 0.99 | 0.87 | 0.68 | 0.47 | 0.19 | 0 |  |
|  | mean ICH | 0.97 | 0.88 | 0.74 | 0.55 | 0.20 | -0.19 | 0 | (32) |
|  | ICH – lower | 0.94 | 0.86 | 0.71 | 0.52 | 0.17 | -0.23 | 0 |  |
|  | ICH - upper | 1.00 | 0.89 | 0.78 | 0.59 | 0.23 | 0.15 | 0 |  |
| EVT MeVO | Intervention | 0.029 | 0.084 | 0.174 | 0.112 | 0.092 | 0.007 | 0 | (29, 33) |
|  | Usual care | 0.010 | 0.056 | 0.108 | 0.099 | 0.126 | 0.013 | 0 |  |
| CASES | Intervention | 0.170 | 0.207 | 0.139 | 0.089 | 0.052 | 0.006 | 0 | (33) |
|  | Usual care | 0.066 | 0.188 | 0.149 | 0.102 | 0.072 | 0.009 | 0 |  |
| ICH | Intervention | 0.021 | 0.113 | 0.141 | 0.117 | 0.032 | -0.020 | 0 | (32) |
|  | Usual care | 0.002 | 0.096 | 0.119 | 0.095 | 0.039 | -0.027 | 0 |  |
| Weighted cost by modified Rankin Scale proportion | | | | | | | | | |
| EVT MeVO | Usual care | €24,724 | | | | | | | (33, 34) |
|  | Intervention | €22,706 | | | | | | |  |
| CASES | Usual care | €22,021 | | | | | | | (29, 34) |
|  | Intervention | €20,136 | | | | | | |  |
| DIST - ICH | Usual care | €34,089 | | | | | | | (32) |
|  | Intervention | €32,164 | | | | | | |  |

Note: ꝉ = Treatment effect

Abbreviations: DIST = Dutch Intracerebral Haemorrhage Surgery Trial, EQ-5D = EuroQol 5 Dimensions, EVT = endovascular treatment, ICH = intracerebral haemorrhage, , LVO = large vessel occlusion, mRS = modified Rankin Scale, MeVO = medium vessel occlusion.

## Key results of qualitative assessment

Question 4 ‘Output’ captured the over-arching theme of the consortia or a combination of certain work packages, followed by Question 5 ‘Outcomes’ specified the changes in behaviour, relationships, actions and activities of stakeholders. It was acknowledged that much of the impact of projects required knowledge utilisation, e.g. uptake of a new therapeutic by clinicians however, this was not possible without for instance, intellectual property rights, funding for future research, or reimbursement, all of which would occur after the funded project period. Therefore, Question 6 ‘Additional steps’ captured the Knowledge Utilisation and Productive Interactions (contact between the researcher and interested target group)(35).

Table 11: consortia - Outcomes

| Outcomes | N | % |
| --- | --- | --- |
| Improve risk stratification in patients | 21 | 66% |
| Provide personalised medicine | 12 | 38% |
| Improve diagnosis in patients | 11 | 34% |
| Identify new biomarkers/pathophysiological mechanism | 11 | 34% |
| Develop new therapeutics | 8 | 25% |
| Prevention of flow-on consequence of CVD | 6 | 19% |
| Changes to infrastructure and methodology | 1 | 3% |
| Improve continuity between care levels (secondary and primary) | 1 | 3% |
| **Sum - Outcome** | **71** | **222^†^%** |

† More than one answer is possible, therefore, the sum is greater than 100%

Table 12: consortia – Additional steps needed to achieve the potential impact

| Additional Step | N | % |
| --- | --- | --- |
| Uptake/implementation of clinical guidelines | 31 | 97% |
| Uptake of new diagnostic/therapeutic by clinicians | 22 | 69% |
| Translation of research towards clinical application | 16 | 50% |
| Intellectual Property rights | 14 | 44% |
| Reimbursement of diagnostic/therapeutic | 13 | 41% |
| Collaborate with industry to facilitate market access | 12 | 38% |
| Acceptance/adherence of new technology by patient | 11 | 34% |
| Acceptance of new technology by family/caregivers | 9 | 28% |
| Public/Private partnerships, e.g. service partners/affiliated businesses | 8 | 25% |
| Training of healthcare providers to use diagnostic or therapeutic | 7 | 22% |
| Changes to legislation and regulation | 6 | 19% |
| Reimbursement of clinician time/consultation | 5 | 16% |
| Future research projects using new database/infrastructure | 3 | 9% |
| Infrastructure adaptation with third-party | 1 | 3% |
| Development of financing structure, e.g. membership fee from patients | 1 | 3% |
| **Sum - additional step** | **159** | **497%**^†^ |

† More than one answer is possible, therefore, the sum is greater than 100%

Table 13: consortia - Impact

| Impact | N | % | Top Ranked |
| --- | --- | --- | --- |
| Improvements in morbidity (e.g. reduction in complications and reliance on surgery) | 31 | 97% | 12 (38%) |
| Improvements in mortality (e.g. reduction in deaths due to heart failure) | 21 | 66% | 12 (38%) |
| Improvements in patients access to care (e.g. convenience) | 11 | 34% | - |
| Appropriate care in patients groups with previously unmet need (e.g. equity in terms of gender or rare-disease) | 8 | 25% | - |
| Improvements in healthcare workforce capacity | 8 | 25% | - |
| Improvements in patient experience/patient satisfaction (e.g. comfort) | 7 | 22% | 4 (13%) |
| Reduction in incidence of CVD | 4 | 13% | 4 (13%) |
| Reduction in costs to the healthcare system | 4 | 13% | - |
| Reduction in research costs | 2 | 6% | - |
| Reduction in productivity losses to the patient | 2 | 6% | - |
| Reduction in waiting time | 1 | 3% | - |
| **Sum - Impact** | **99** | **309%^†^** | **100%** |

† More than one answer is possible, therefore, the sum is greater than 100%

## Key results of quantitative assessment

The sensitivity analysis used draws from independent normal distributions for all parameters involved, the analysis was repeated 1,000 times, and then the 95%CI was calculated.

A summary of findings regarding health benefits is presented below:

- The analysis of the Check@Home consortium indicated how a home-based screening could reduce the risk of CVD (both atrial fibrillation and heart failure), CKD and diabetes. It is expected that there will be 212 (56+55+86+20) QALYs gained in the first year with potential cost savings.
- The analysis of the LoDoCo2 consortium indicated how a repurposed drug can be expected to treat CAD and result in 362 QALYs gained based on a lifetime benefit from the published economic evaluation (36). Assuming the modest cost of colchicine, this could also lead to cost savings.
- The analysis of the IMPRESS consortium consisted of four sub-studies of women with CAD (including non‑obstructive CAD) and the potential health benefits. The first sub-study could contribute to additional life years (LYs) due to an earlier diagnosis and the second sub‑study could lead to a QALY improvement from testing with invasive coronary angiography during treatment for MACE events (173 and 435 QALYs, respectively). The remaining two sub‑studies did not produce a quantifiable health benefit.
- The analysis of the CONTRAST 2.0 consortium consisted of three sub‑studies of patients with stroke, resulting in QALYs in Year 1, 191, 51 and 106, respectively.

Table 14: Quantitative Impact Monitor – Summary – Check@Home

|  |  | CVD-AF | CVD-HF | CKD | Diabetes |
| --- | --- | --- | --- | --- | --- |
| Annual number of patients in the Netherlands that will receive the intervention | Prevalence | 5,694,301 | 5,694,301 | 5,694,301 | 5,694,301 |
|  | Screened | 965,487 | 965,487 | 965,487 | 965,487 |
|  | Number of patients | 53,102 | 17,211 | 144,823 | 80,790 |
| Estimate the potential effect on health from the intervention (compared to usual care). Use primary outcome measure in study. | Primary outcome | 1year prob. of IS event | 1-year prob. of HF event | 1-year prob. of KF | - |
|  | Effect of usual care | 0.037 | 0.217 | 0.066 | - |
|  | Effect of intervention | 0.011 | 0.100 | 0.041 | - |
|  | Potential effect [intervention minus usual care] | -0.027 | -0.117 | -0.025 | - |
|  | Effect of intervention – lower | 0.011 | 0.107 | 0.046 | - |
|  | Effect of intervention - upper | 0.010 | 0.077 | 0.041 | - |
| Estimate the potential effect on resource utilisation (compared to usual care). Report number of events. | Event | IS event | HF event | KF event | HF event |
|  | Effect of usual care | 1,978 | 3,739 | 9,491 | 435 |
|  | Effect of intervention | 616 | 1,893 | 6,532 | 220 |
|  | Potential effect [intervention minus usual care] | -1,362 | -837 | -2,959 | -215 |
|  | Effect of intervention – lower | 619 | 2,033 | 7,301 | 236 |
|  | Effect of intervention - upper | 582 | 1,450 | 6,456 | 206 |
| Estimate the potential effect on health from the intervention (compared to usual care). Report QALYs. | Effect of usual care | - | -202 | -550 | -80 |
|  | Effect of intervention | - | -102 | -379 | -40 |
|  | Potential effect [intervention minus usual care] | 113 | 100 | 172 | 39 |
|  | Effect of intervention – lower | 113 | -110 | -423 | -44 |
|  | Effect of intervention - upper | 116 | -78 | -374 | -38 |
| Estimate the potential effects on costs (€) associated with the intervention (compared to usual care), annual cost per patient | Cost of usual care | € 0 | € 0 | € 0 | € 0 |
|  | Cost of intervention | € 7 | € 7 | € 7 | € 7 |
|  | Cost of usual care consequences | € 41,998 | € 8,826 | € 117,996 | € 8,826 |
|  | Cost of intervention consequences | € 44,469 | € 8,826 | € 117,996 | € 8,826 |
| Estimate the potential effects on costs (€), annual cost, total treated | Cost of usual care | € 0 | € 0 | € 0 | € 0 |
|  | Cost of intervention | € 6,436,580 | € 6,436,580 | € 6,436,580 | € 6,436,580 |
|  | Cost of usual care consequences | € 83,051,812 | € 33,004,494 | € 1,119,850,222 | € 3,836,226 |
|  | Cost of intervention consequences | € 27,377,719 | €16,709,388 | € 770,694,570 | € 1,942,190 |
| Estimate the potential cost-savings, corrected for implementation | Potential cost-savings of new intervention | € 49,237,512 | € 9,858,526 | € 342,719,072 | -€ 4,542,554 |
|  | % implementation | 50% | 50% | 50% | 50% |
|  | Potential cost-savings (implementation) | € 24,618,756 | € 4,929,263 | € 171,359,536 | -€ 2,271,272 |
| Estimate the potential additional QALY benefit for total treated, corrected for implementation | Potential additional benefit in QALY | 113 | 100 | 172 | 39 |
|  | % implementation | 50% | 50% | 50% | 50% |
|  | Potential additional benefit in QALY (implementation) | 56 | 50 | 86 | 20 |
|  | Potential additional benefit in QALY (implementation) - lower | 56 | 46 | 63 | 18 |
|  | Potential additional benefit in QALY (implementation) - upper | 58 | 62 | 88 | 21 |

Abbreviations: AF = atrial fibrillation, CKD = chronic kidney disease, CVD = cardiovascular disease, HF = heart failure, IS = ischaemic stroke, KF= kidney failure, QALYs = quality adjusted life year

Table 15: Quantitative Impact Monitor – Summary – LoDoCo2

|  |  | LoDoCo2 |
| --- | --- | --- |
|  |  | CAD |
| Annual number of patients in the Netherlands that will receive the intervention | Prevalence | 847,912 |
|  | Incidence | 14,464 |
|  | Number of patients | 10,125 |
|  | Primary outcome | MACE event |
| Estimate the potential effect on health from the intervention (compared to usual care). Use primary outcome measure in study. | Effect of usual care | 0.096 |
|  | Effect of intervention | 0.068 |
|  | Potential effect [intervention minus usual care] | -0.028 |
|  | Effect of intervention – lower | 0.055 |
|  | Effect of intervention - upper | 0.080 |
| Estimate the potential effect on resource utilisation (compared to usual care). Report number of events. | Event | MACE events |
|  | Effect of usual care | 972 |
|  | Effect of intervention | 688 |
|  | Potential effect [intervention minus usual care] | -283 |
|  | Effect of intervention – lower | 554 |
|  | Effect of intervention - upper | 807 |
| Estimate the potential effect on health from the intervention (compared to usual care). Report QALYs. | Effect of usual care | - |
|  | Effect of intervention | - |
|  | Potential effect [intervention minus usual care] | 0.04^†^ |
|  | Effect of intervention – lower | 0.04 |
|  | Effect of intervention - upper | 0.04 |
|  | Cost of usual care | € 0 |
| Estimate the potential effects on costs (€) associated with the intervention (compared to usual care), annual cost per patient | Cost of intervention | € 113 |
|  | Cost of usual care consequences | € 51,294 |
|  | Cost of intervention consequences | € 51,294 |
|  | Cost of usual care | € 0 |
| Estimate the potential effects on costs (€), annual cost, total treated | Cost of intervention | € 1,144,102 |
|  | Cost of usual care consequences | € 49,857,209 |
|  | Cost of intervention consequences | € 35,315,523 |
|  | Potential cost-savings of new intervention | € 13,397,583 |
| Estimate the potential cost-savings, corrected for implementation | % implementation | 90% |
|  | Potential cost-savings (implementation) | € 11,990,837 |
|  | Potential additional benefit in QALY | 405 |
| Estimate the potential additional QALY benefit for total treated, corrected for implementation | % implementation | 90% |
|  | Potential additional benefit (implementation) | 362 |
|  | Potential additional benefit in QALY (implementation) - lower | 355 |
|  | Potential additional benefit in QALY (implementation) - upper | 400 |

† Lifetime per patient.

Abbreviations: CAD = coronary artery disease, ICA = invasive coronary angiography, MACE = myocardial infarction, stroke, or cardiovascular death, with or without coronary revascularisation (PCI or CABG), MVA = microvascular angina, QALY = quality adjusted life year

Table 16: Quantitative Impact Monitor – Summary – IMPRESS

|  |  | IMPRESS | IMPRESS | IMPRESS | IMPRESS |
| --- | --- | --- | --- | --- | --- |
|  |  | CAD - Women with under-diagnosed CAD | CAD - Women with non-obstructive with ICA | CAD - Women with non-obstructive ICA vs. non-invasive imaging | CAD -women with non-obstructive, MVA phenotype |
| Annual number of patients in the Netherlands that will receive the intervention | Prevalence | 426,360 | - | - | - |
|  | Incidence | 7,273 | - | - | - |
|  | Number of patients | 7,273 | 4,582 | 4,582 | 2,367 |
|  | Primary outcome | Diagnosis of CVD | MACE events for ICA vs. no testing | MACE events | Successful treatment |
| Estimate the potential effect on health from the intervention (compared to usual care). Use primary outcome measure in study. | Effect of usual care | 7,273 | - | - | 0.29 |
|  | Effect of intervention | 7,273 | - | - | 0.21 |
|  | Potential effect [intervention minus usual care] | 0 | 0 | 0 | -0.08 |
|  | Effect of intervention – lower | - | - | - | - |
|  | Effect of intervention - upper | - | - | - | - |
| Estimate the potential effect on resource utilisation (compared to usual care). Report number of events. | Event | Healthcare resource use | MACE events | MACE events | Epicardial spasm |
|  | Effect of usual care | - | 119 | 170 | 0.54 |
|  | Effect of intervention | - | 119 | 170 | 0.32 |
|  | Potential effect [intervention minus usual care] | - | 0 | 0 | -0.23 |
|  | Effect of intervention – lower | - | - | - | - |
|  | Effect of intervention - upper | - | - | - | - |
| Estimate the potential effect on health from the intervention (compared to usual care). Report QALYs. | Effect of usual care | 7.40^‡†^ | 0.50 | - | - |
|  | Effect of intervention | 7.43^‡†^ | 0.66 | - | - |
|  | Potential effect [intervention minus usual care] | 0.03^‡†^ | 0.10 | 0 | 0 |
|  | Effect of intervention – lower | 7.42 | 0.10 | - | - |
|  | Effect of intervention - upper | 7.43 | 0.10 | - | - |
|  | Cost of usual care | € 0 | € 0 | € 1,869 | € 0 |
| Estimate the potential effects on costs (€) associated with the intervention (compared to usual care), annual cost per patient | Cost of intervention | € 20 | € 1,869 | € 244 | € 14 |
|  | Cost of usual care consequences | € 10,902 | € 51,294 | € 51,294 | - |
|  | Cost of intervention consequences | € 10,902 | € 51,294 | € 51,294 | - |
|  | Cost of usual care | € 0 | € 0 | € 8,564,602 | € 0 |
| Estimate the potential effects on costs (€), annual cost, total treated | Cost of intervention | € 145,460 | € 8,564,602 | € 1,118,743 | € 32,000 |
|  | Cost of usual care consequences | € 79,287,583 | € 235,030,736 | € 235,030,736 | € 0 |
|  | Cost of intervention consequences | € 79,287,583 | € 235,030,736 | € 235,030,736 | € 0 |
|  | Potential cost-savings of new intervention | -€ 145,460 | -€ 8,564,602 | € 7,445,858 | -€ 32,000 |
| Estimate the potential cost-savings, corrected for implementation | % implementation | 95% | 95% | 95% | 95% |
|  | Potential cost-savings (implementation) | -€ 138,187 | -€ 8,136,372 | € 7,073,566 | -€ 30,400 |
|  | Potential additional benefit in QALY | 182^‡^ | 458 | 0 | 0 |
| Estimate the potential additional QALY benefit for total treated, corrected for implementation | % implementation | 95% | 95% | 95% | 95% |
|  | Potential additional benefit (implementation) | 173^‡^ | 435 | 0 | 0 |
|  | Potential additional benefit in QALY (implementation) - lower | 157 | 421 | 0 | 0 |
|  | Potential additional benefit in QALY (implementation) - upper | 179 | 448 | 0 | 0 |

‡ Survival reported in life years and not quality adjusted life years.

Abbreviations: CAD = coronary artery disease, ICA = invasive coronary angiography, MACE = myocardial infarction, stroke, or cardiovascular death, with or without coronary revascularisation (PCI or CABG), MVA = microvascular angina, QALY = quality adjusted life year

Table 17: Quantitative Impact Monitor – Summary –CONTRAST 2.0

|  |  | EVT in MeVO | Immediate CAS LVO | Minimally invasive surgery for ICH |
| --- | --- | --- | --- | --- |
| Annual number of patients in the Netherlands that will receive the intervention | Prevalence | - | - | - |
|  | Incidence | 9,424 | 9,424 | 9,424 |
|  | Number of patients | 3,016 | 707 | 1,414 |
|  | Primary outcome | mRS at 3 months | mRS at 3 months | mRS at 180 days |
| Estimate the potential effect on health from the intervention (compared to usual care). Use primary outcome measure in study. | Effect of usual care | 4 | 0 | 0 |
|  | Effect of intervention | 3 | 0 | 0 |
|  | Potential effect [intervention minus usual care] | 1.67 | 1.15 | 1.49 |
|  | Effect of usual care | 0.41 | 0.58 | 0.32 |
|  | Effect of intervention | 0.50 | 0.66 | 0.40 |
| Estimate the potential effect on health from the intervention (compared to usual care). Report QALYs. | Potential effect [intervention minus usual care] | 0.07 | 0.08 | 0.08 |
|  | Effect of intervention – lower | 0.46 | 0.65 | 0.39 |
|  | Effect of intervention - upper | 0.52 | 0.68 | 0.42 |
|  | Cost of usual care | € 0 | € 6,188 | € 0 |
|  | Cost of intervention | € 11,430 | € 8,483 | € 11,976 |
| Estimate the potential effects on costs (€) associated with the intervention (compared to usual care), annual cost per patient | Cost of usual care consequences | € 24,724 | € 22,021 | € 34,089 |
|  | Cost of intervention consequences | € 22,706 | € 20,136 | € 32,164 |
|  | Cost of usual care | € 0 | € 4.373.754 | € 0 |
|  | Cost of intervention | € 34,470,403 | € 5,995,924 | € 16,929,644 |
| Estimate the potential effects on costs (€), annual cost, total treated | Cost of usual care consequences | € 74,559,405 | € 15,564,376 | € 48,188,513 |
|  | Cost of intervention consequences | € 68,473,091 | € 14,232,046 | € 45,466,962 |
|  | Potential cost-savings of new intervention | -€ 28,384,089 | -€ 289,840 | -€ 14,208,093 |
|  | % implementation | 93% | 93% | 93% |
| Estimate the potential cost-savings, corrected for implementation | Potential cost-savings (implementation) | -€ 26,313,147 | -€ 268,693 | -€ 13,171,451 |
|  | Potential additional benefit in QALY | 206 | 55 | 114 |
|  | % implementation | 93% | 93% | 93% |
| Estimate the potential additional QALY benefit for total treated, corrected for implementation | Potential additional benefit (implementation) | 191 | 51 | 106 |
|  | Potential additional benefit in QALY (implementation) - lower | 177 | 42 | 91 |
|  | Potential additional benefit in QALY (implementation) - upper | 236 | 61 | 123 |

Abbreviations: CAS = carotid artery stenting, EVT = endovascular treatment, ICH = intracerebral haemorrhage, LVO = large vessel occlusion, MeVO = medium vessel occlusion, mRS = modified Rankin Scale, QALY = quality adjusted life year.

Table 18: Estimation of QALYs from the four projects of the DCVA consortia

|  | | Additional QALY Year 1 | | Additional QALYs Lifetime | | Total QALYs (2023 only) | | Total QALYs (cumulative to 2030), | |
| --- | --- | --- | --- | --- | --- | --- | --- | --- | --- |
|  | | Base-case estimate | | | | | | | |
| Check@Home | | 212[56+ 50+86+20] | | - | | 212 | | 1,694 | |
| LoDoCo2 | | - | | 362 | | 362 | | 362 | |
| IMPRESS | | 435 | | 173^†^ | | 608 | | 3,655 | |
| CONTRAST 2.0 | | 348 [191+51+106] | | - | | 348 | | 2,783 | |
| Impact of 4 Projects | | 995 | | 535 | | 1,530 | | 8,494 | |
| **Impact of 32 Projects** | | **67,954** | | | | | | | |
|  | | Lower estimate | | | | | | | |
| Check@Home | | 184 [56 + 46 +63 + 18] | | - | | 184 | | 1,471 | |
| LoDoCo2 | | - | | 355 | | 355 | | 355 | |
| IMPRESS | | 421 | | 157^†^ | | 578 | | 3,463 | |
| CONTRAST 2.0 | | 309 [177+42+91] | | - | | 309 | | 2,643 | |
| Impact of 4 Projects | | 2,065 | | 512 | | 1,426 | | 8,042 | |
| **Impact of 32 Projects** | | **62,580** | | | | | | | |
|  | | Upper estimate | | | | | | | |
| Check@Home | | 229 [58 + 62 + 88 + 21] | | - | | 229 | | 1,829 | |
| LoDoCo2 | | - | | 400 | | 400 | | 400 | |
| IMPRESS | | 448 | | 179^†^ | | 627 | | 3,764 | |
| CONTRAST 2.0 | | 420 [236 + 61 + 123] | | - | | 420 | | 3,363 | |
| Impact of 4 Projects | | 1,097 | | 579 | | 1,676 | | 9,356 | |
| **Impact of 32 Projects** | | **74,844** | | | | | | | |

^†^Life years. ‡ Implementation refers to the adherence rate assumed in the analyses, i.e. Check@Home = 50% [Table 12], LoDoCo2 = 90% [Table 13], IMPRESS = 95%.[Table 14], CONTRAST 2.0 = 93% [Table 15

Disability adjusted life years (DALYs) were used to quantify disease burden. DALYs are a societal measure of disease or disability and are estimated as the sum of years of life lost (YLLs) to reflect premature mortality and the years of healthy life lost due to disability (YLDs)(37). If the condition is likely fatal, then it can be assumed that QALYs are equivalent to DALYs(37). Given the substantial risk that a severe CVD event is fatal, DALYs and QALYs were assumed to be equivalent in the analysis. A summary of the main steps taken to 1) estimate the 2030 CVD burden in the Netherlands, and 25% of that burden, and 2) estimate how all DCVA consortia combined are expected to contribute to achieving the 25% burden reduction, is presented in Box 2.

Table 19: Steps to estimate the current burden of CVD in the Netherlands and the absolute value of a 25% reduction in CVD burden

1. Estimate the CVD burden in the Netherlands in DALYs, over the period 2023-2030, based on the estimated DALYs in 2023. Assume the QALY loss equals DALY loss.
2. Calculate the QALYs that need to be gained to reduce the CVD burden by 25%.
3. Estimate the QALY-based burden reduction from the DCVA consortia (from Part B).
4. Estimate the average QALY gain per person based on the quantitative assessment of the four consortia.
   1. Sum the additional QALYs gained (accounting for implementation) from all four consortia and taking into consideration estimations for Year 1 (2023) and estimations over a lifetime of individuals benefiting from the intervention within the consortia.
   2. Estimate the accumulated QALYs until 2030, i.e. over a 7-year period. Taking into account the annual or lifetime estimation from the quantitative assessment, i.e. if annual QALY gain was estimated then this can be multiplied to obtain QALY estimates over a 7-year period, but this is not possible if a lifetime estimation was previously conducted in the analysis in Section 2 of the Report.
   3. Extend the sample to the entire DCVA portfolio of consortia by multiplying the estimated value from Step 3b by eight (32 consortia divided by four).
5. Estimate the reduction in CVD burden from the impact of the DCVA consortia.
6. Divide the average QALY gain from Step 3c by the average absolute QALY (burden of CVD) from Step 2 to obtain the proportion of the QALY gain from DCVA consortia contributing to the aim of the DCVA to reduce CVD burden.

Abbreviations: CVD = cardiovascular disease, DALYs = disability adjusted life years, DCVA = Dutch Cardiovascular Alliance, QALYs = quality adjusted life years.

Table 20: Estimation of the reduction in CVD burden from the impact of the DCVA consortia

|  | Description | Value | Reference |
| --- | --- | --- | --- |
| a | Total DALYs from CVD in the Netherlands in 2023 to 2030 | 348,490 | (38) and calculated |
| b | Goal 25% reduction | 25% | DCVA |
| c | Absolute DALY/QALY to equate 25% reduction^†^ | 87,122 | a*b |
| d | Total additional QALY gain in 2023 to 2030 | 67,954 | Table 17 |
|  | Proportion of DCVA consortia contributing to DCVA aim of CVD burden reduction | 78% | d/c |
| e | Total additional QALY gain in 2023 to 2030 – lower estimate | 62,580 | Table 17 |
|  | Proportion of DCVA consortia contributing to DCVA aim of CVD burden reduction – lower | 72% | e/c |
| f | Total additional QALY gain in 2023 to 2030 – upper estimate | 74,844 | Table 17 |
|  | Proportion of DCVA consortia contributing to DCVA aim of CVD burden reduction – upper | 86% | f/c |

†Assume DALYs are equivalent to QALYs due to potentially fatal-condition(37).

The CVD burden in the Netherlands was measured in terms of DALYs. This resulted in an estimated total burden from CVD of 348,490 DALYs(38) over the years 2023-2030 [grey line] (see Figure 3). Next, the target 25% reduction in CVD burden was calculated to be 87,122 DALYs (25% of 348,490). Consequently, the DCVA ambition is to limit the DALYs over the years 2023-2030 to 261,368 DALYs (348,490 – 87,122 DALYs, the orange line).

Then, the QALYs expected to be gained from the four case‑studies from Part B were linearly extrapolated to estimate the expected potential accumulated health benefit of all 32 consortia from 2023-2030. This extrapolation indicated that 67,954 QALYs would be gained from all 32 consortia. Under the assumption that the QALY loss from CVD would be equivalent to the DALYs, the growth in DALYs is estimated to be reduced to 282,536 (348,490 – 67,954, red line). Finally, if the 32 consortia would reduce DALYs by 67,954, then 78% of the target aim of reducing the CVD disease burden by 87,122 DALYs (= 25%) is achieved (67,954 / 87,122 DALYs). A sensitivity analysis resulted in a range of 72% to 86%.

Table 21: Accumulated DALYs burden and QALYs benefits from DCVA consortia between 2023 and 2030


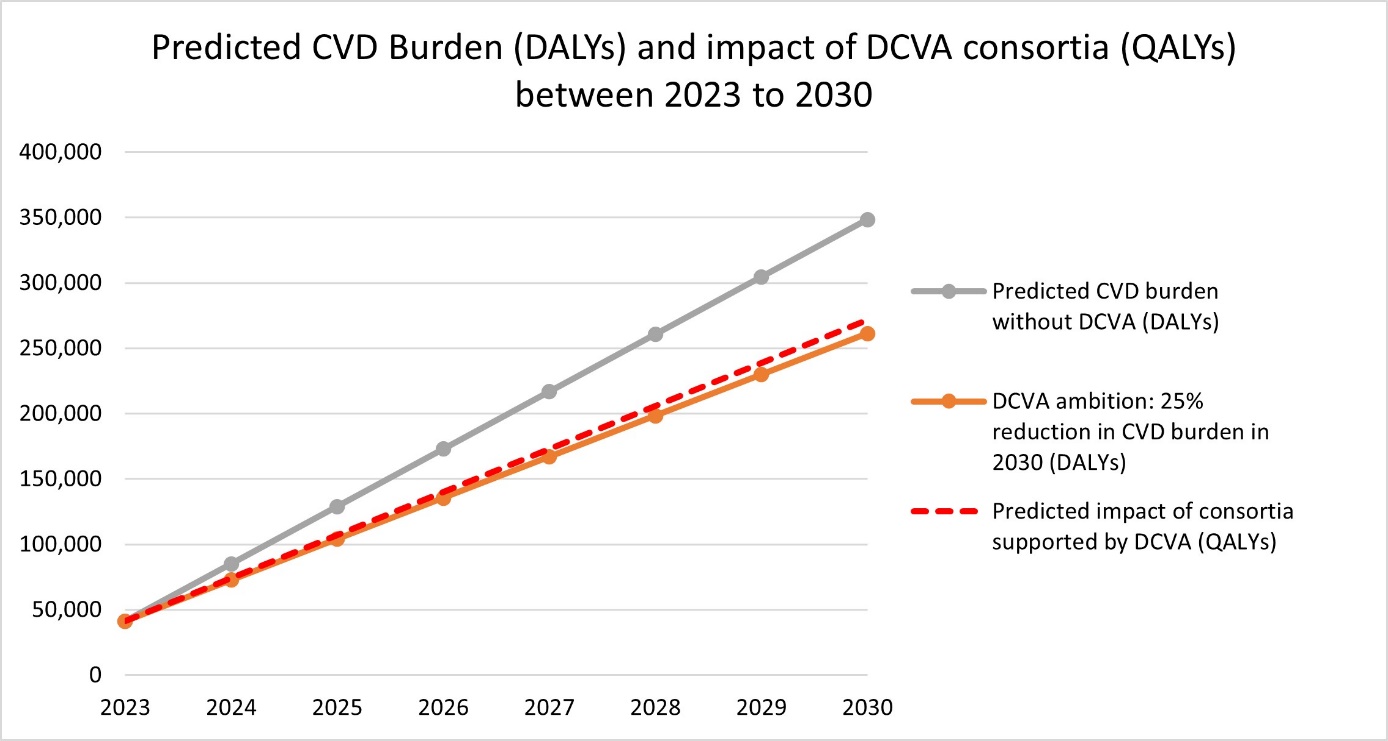


Abbreviations: CVD = cardiovascular disease, DALYS = disability adjusted life years, DCVA = Dutch Cardiovascular Alliance, QALYs = quality adjusted life years.

# References

1. Jacobs MS, Kaasenbrood F, Postma MJ, van Hulst M, Tieleman RG. Cost-effectiveness of screening for atrial fibrillation in primary care with a handheld, single-lead electrocardiogram device in the Netherlands. Europace. 2018;20(1):12-8.

2. Parveen S, Zareini B, Arulmurugananthavadivel A, Kistorp C, Faber J, Køber L, et al. Association between early detected heart failure stages and future cardiovascular and non-cardiovascular events in the elderly (Copenhagen Heart Failure Risk Study). BMC Geriatrics. 2022;22(1):230.

3. Burnett H, Earley A, Voors AA, Senni M, McMurray JJ, Deschaseaux C, et al. Thirty Years of Evidence on the Efficacy of Drug Treatments for Chronic Heart Failure With Reduced Ejection Fraction: A Network Meta-Analysis. Circ Heart Fail. 2017;10(1):e003529.

4. Grams ME, Yang W, Rebholz CM, Wang X, Porter AC, Inker LA, et al. Risks of Adverse Events in Advanced CKD: The Chronic Renal Insufficiency Cohort (CRIC) Study. Am J Kidney Dis. 2017;70(3):337-46.

5. Xie X, Liu Y, Perkovic V, Li X, Ninomiya T, Hou W, et al. Renin-Angiotensin System Inhibitors and Kidney and Cardiovascular Outcomes in Patients With CKD: A Bayesian Network Meta-analysis of Randomized Clinical Trials. Am J Kidney Dis. 2016;67(5):728-41.

6. Groenewegen A, Zwartkruis VW, Cekic B, de Boer RA, Rienstra M, Hoes AW, et al. Incidence of atrial fibrillation, ischaemic heart disease and heart failure in patients with diabetes. Cardiovasc Diabetol. 2021;20(1):123.

7. Baeten SA, van Exel NJ, Dirks M, Koopmanschap MA, Dippel DW, Niessen LW. Lifetime health effects and medical costs of integrated stroke services - a non-randomized controlled cluster-trial based life table approach. Cost Eff Resour Alloc. 2010;8:21.

8. McMurray JJV, Trueman D, Hancock E, Cowie MR, Briggs A, Taylor M, et al. Cost-effectiveness of sacubitril/valsartan in the treatment of heart failure with reduced ejection fraction. Heart. 2018;104(12):1006-13.

9. Li B, Cairns JA, Draper H, Dudley C, Forsythe JL, Johnson RJ, et al. Estimating Health-State Utility Values in Kidney Transplant Recipients and Waiting-List Patients Using the EQ-5D-5L. Value Health. 2017;20(7):976-84.

10. Keng MJ, Leal J, Bowman L, Armitage J, Mihaylova B. Decrements in health-related quality of life associated with adverse events in people with diabetes. Diabetes Obes Metab. 2022;24(3):530-8.

11. Sülz S, Wagenaar H, van de Klundert J. Have Dutch Hospitals Saved Lives and Reduced Costs? A longitudinal patient-level analysis over the years 2013–2017. Health Economics. 2021;30(10):2399-408.

12. Mohnen SM, van Oosten MJM, Los J, Leegte MJH, Jager KJ, Hemmelder MH, et al. Healthcare costs of patients on different renal replacement modalities - Analysis of Dutch health insurance claims data. PLoS One. 2019;14(8):e0220800.

13. nierpatientien vereniging nederland. Jaarcijfers orgaandonatie 2022 laten zien: nieuwe Donorwet is succesvol [Available from: <https://www.nvn.nl/nieuws/jaarcijfers-orgaandonatie-2022-laten-zien-nieuwe-donorwet-is-succesvol/>.

14. nefrovisie. Jaarcijfers Renine 2020 [Available from: <https://www.nefrovisie.nl/nieuwsbrief/jaarcijfers-renine-2020/#:~:text=Het%20aantal%20pati%C3%ABnten%20met%20nierfunctievervangende,pati%C3%ABnten%20worden%20met%20dialyse%20behandeld>.

15. van Exel NJ, Koopmanschap MA, Scholte op Reimer W, Niessen LW, Huijsman R. Cost-effectiveness of integrated stroke services. QJM. 2005;98(6):415-25.

16. Nidorf SM, Fiolet ATL, Mosterd A, Eikelboom JW, Schut A, Opstal TSJ, et al. Colchicine in Patients with Chronic Coronary Disease. New England Journal of Medicine. 2020;383(19):1838-47.

17. Burger PM, Dorresteijn JAN, Fiolet ATL, Koudstaal S, Eikelboom JW, Nidorf SM, et al. Individual lifetime benefit from low-dose colchicine in patients with chronic coronary artery disease. European Journal of Preventive Cardiology. 2023:zwad221.

18. Fiolet and Keusters et al. Cost-effectiveness of low-dose colchicine in patients with chronic coronary disease. Submitted. 2023.

19. Berkelmans GFN, Greving JP, van der Graaf Y, Visseren FLJ, Dorresteijn JAN. Would treatment decisions about secondary prevention of CVD based on estimated lifetime benefit rather than 10-year risk reduction be cost-effective? Diagn Progn Res. 2020;4:4.

20. Crimmins EM, Hayward MD, Ueda H, Saito Y, Kim JK. Life with and without heart disease among women and men over 50. J Women Aging. 2008;20(1-2):5-19.

21. Ford TJ, Stanley B, Good R, Rocchiccioli P, McEntegart M, Watkins S, et al. Stratified Medical Therapy Using Invasive Coronary Function Testing in Angina: The CorMicA Trial. J Am Coll Cardiol. 2018;72(23 Pt A):2841-55.

22. Peper J, Becker LM, Bruning TA, Budde RPJ, van Dockum WG, Frederix GWJ, et al. Rationale and design of the iCORONARY trial: improving the cost-effectiveness of coronary artery disease diagnosis. Neth Heart J. 2023;31(4):150-6.

23. Jansen TPJ, Konst RE, de Vos A, Paradies V, Teerenstra S, van den Oord SCH, et al. Efficacy of Diltiazem to Improve Coronary Vasomotor Dysfunction in ANOCA: The EDIT-CMD Randomized Clinical Trial. JACC: Cardiovascular Imaging. 2022;15(8):1473-84.

24. Darlington M, Gueret P, Laissy JP, Pierucci AF, Maoulida H, Quelen C, et al. Cost-effectiveness of computed tomography coronary angiography versus conventional invasive coronary angiography. Eur J Health Econ. 2015;16(6):647-55.

25. Zorginstituutnederland. Medicijnkosten.nl [Available from: <https://www.medicijnkosten.nl/>.

26. Shaw LJ, Merz CN, Pepine CJ, Reis SE, Bittner V, Kip KE, et al. The economic burden of angina in women with suspected ischemic heart disease: results from the National Institutes of Health--National Heart, Lung, and Blood Institute--sponsored Women's Ischemia Syndrome Evaluation. Circulation. 2006;114(9):894-904.

27. Wang ZJ, Zhang LL, Elmariah S, Han HY, Zhou YJ. Prevalence and Prognosis of Nonobstructive Coronary Artery Disease in Patients Undergoing Coronary Angiography or Coronary Computed Tomography Angiography: A Meta-Analysis. Mayo Clin Proc. 2017;92(3):329-46.

28. Singh N, Bala F, Kashani N, Horn M, Stang J, Demchuk AM, et al. Prediction of 90‐Day Home Time Among Patients With Medium‐Vessel Occlusion Undergoing Endovascular Thrombectomy. Stroke: Vascular and Interventional Neurology. 2023;3(4):e000748.

29. University Medical Center Groningen. Carotid Artery Stenting during Endovascular treatment for Stroke (CASES) Protocol Version 2.3.

30. van Voorst H, Kunz WG, van den Berg LA, Kappelhof M, Pinckaers FME, Goyal M, et al. Quantified health and cost effects of faster endovascular treatment for large vessel ischemic stroke patients in the Netherlands. J Neurointerv Surg. 2021;13(12):1099-105.

31. Janssen MP, de Borst GJ, Mali WPTM, Kappelle LJ, Moll FL, Ackerstaff RGA, et al. Carotid Stenting versus Carotid Endarterectomy: Evidence Basis and Cost Implications. European Journal of Vascular and Endovascular Surgery. 2008;36(3):258-64.

32. Schreuder F, Scholte M, Ulehake MJ, Sondag L, Rovers MM, Dammers R, et al. Identifying the Conditions for Cost-Effective Minimally Invasive Neurosurgery in Spontaneous Supratentorial Intracerebral Hemorrhage. Front Neurol. 2022;13:830614.

33. Dijkland; SA, Voormolen; DC, Venema; E, Roozenbeek; B, Polinder; S, Haagsma; JA, et al. Utility-Weighted Modified Rankin Scale as Primary Outcome in Stroke Trials. Stroke. 2018;49(4):965-71.

34. Wilson A, Bath PM, Berge E, Cadilhac DA, Cuche M, Ford GA, et al. Understanding the relationship between costs and the modified Rankin Scale: A systematic review, multidisciplinary consensus and recommendations for future studies. Eur Stroke J. 2017;2(1):3-12.

35. Dutch Research Council (NWO). Definitions knowledge utilisation: NWO; 2024 [Available from: <https://www.nwo.nl/en/definitions-knowledge-utilisation>.

36. Fiolet ATL, Keusters W, Blokzijl J, Nidorf SM, Eikelboom JE, Budgeon CA, et al. Cost-effectiveness of low-dose colchicine in patients with chronic coronary disease in the netherlands. Eur Heart J Qual Care Clin Outcomes. 2024.

37. Sassi F. Calculating QALYs, comparing QALY and DALY calculations. Health Policy Plan. 2006;21(5):402-8.

38. Hilderink HBM, Plasmans MHD, Poos MJJC, Eysink PED, Gijsen R. Dutch DALYs, current and future burden of disease in the Netherlands. Arch Pub Health. 2020;78(1):85.
